# Mothers exhibit higher neural activity in gaining rewards for their children than for themselves

**DOI:** 10.1093/scan/nsad048

**Published:** 2023-09-13

**Authors:** Yan Zhang, Yachao Rong, Ping Wei

**Affiliations:** Beijing Key Laboratory of Learning and Cognition, School of Psychology, Capital Normal University, Beijing 100048, China; Beijing Key Laboratory of Learning and Cognition, School of Psychology, Capital Normal University, Beijing 100048, China; Beijing Key Laboratory of Learning and Cognition, School of Psychology, Capital Normal University, Beijing 100048, China

**Keywords:** reward, self, child, electroencephalogram (EEG), source localization

## Abstract

Are people willing to exert greater effort to obtain rewards for their children than they are for themselves? Although previous studies have demonstrated that social distance influences neural responses to altruistic reward processing, the distinction between winning rewards for oneself and winning them for one’s child is unclear. In the present study, a group of 31 mothers performed a monetary incentive delay task in which cue-induced reward anticipations of winning a reward for themselves, their children and donation to a charity program were manipulated trial-wise, followed by performance-contingent feedback. Behaviorally, the anticipation of winning a reward for their children accelerated participants’ responses. Importantly, the electroencephalogram results revealed that across the reward anticipation and consumption phases, the child condition elicited comparable or higher brain responses of participants than the self condition did. The source localization results showed that participants’ reward anticipations for their children were associated with more activation in the social brain regions, compared to winning a reward for themselves or a charity donation. Overall, these findings advance our understanding of the neural mechanisms of altruistic reward processing and suggest that the priority of winning a reward for one’s child may transcend the limits of the self-advantage effect in reward processing.

## Introduction

Studies have consistently reported that the neural responses of participants are stronger when they gain rewards for themselves than when they gain them for others, including friends, antagonists and charity programs (Leng and Zhou, 2014; San Martín *et al.*, 2016; Zhu *et al.*, 2016a,b, 2018, 2020; Cui *et al.*, 2018; Luo *et al.*, 2019, 2022; Kwak *et al.*, 2020; Tang *et al.*, 2021; Tan *et al.*, 2022). The level of the brain’s responses has been found to be influenced by the social distance between the beneficiary and participant: a closer social distance between them is associated with stronger brain responses of participants in gaining rewards for others. Compared to winning rewards for a stranger or antagonist, winning rewards for a closely related individual, such as one’s mother or a friend, is associated with stronger brain activity in the ventral striatum, similar to the brain activity observed in persons gaining rewards for themselves (Mobbs *et al.*, 2009; Braams and Crone, 2017a,b; Brandner *et al.*, 2020, 2021).

Reward processing is a dynamic set of actions that can be divided into anticipation and consumption phases. The monetary incentive delay (MID; Knutson *et al.*, 2000; Lutz and Widmer, 2014) paradigm is useful for separating these two stages, in which a cue indicating a reward condition and reward beneficiary is followed by a task requiring a fast response and then by feedback indicating whether the designated beneficiary gained the reward. A study using this paradigm and electrophysiological recording reported a greater cue-related P3 in participants winning a reward for themselves rather than winning one for a charity donation during the reward anticipation phase (Luo *et al.*, 2019). Except for the MID task, the social gambling task (SGT), in which participants choose between ‘decks’ (i.e. stimuli with different probabilities of payoffs for beneficiaries), after which they are presented with gain or loss feedback, is often used to investigate the brain’s response when a rewarding outcome is revealed. San Martín *et al.* reported greater delta (loss minus win) feedback-related negativity (FRN) when participants win for themselves, compared with wins for charity donations during the consumption phase (San Martín *et al.*, 2016). The term reward positivity (RewP) has been suggested recently as a better characterization of the functional significance following gain-related feedback than FRN (Holroyd *et al.*, 2008, 2011; Foti *et al.*, 2011; Proudfit, 2015; Meadows *et al.*, 2016; Paul *et al.*, 2020); however, the joint use of both terms (FRN/RewP) is preferred by other researchers in the field (Krigolson, 2018). Moreover, the feedback-related P3 was also larger for wins for oneself than wins for a friend (Leng and Zhou, 2010; Kwak *et al.*, 2020) or a charity donation (San Martín *et al.*, 2016). Furthermore, several neuroimaging studies have identified a network of brain areas related to thinking about others in reward processing (Van Overwalle, 2009; Braams *et al.*, 2014b), which are also referred to as ‘social brain regions’ (Frith, 2007; Braams *et al.*, 2014a; Braams and Crone, 2017a). Specifically, tasks that required mentalizing, empathizing or thinking about friends or significant others activated the temporo-parietal junction (TPJ), precuneus and ventral medial prefrontal cortex (mPFC; Güroǧlu *et al.*, 2008; Braams and Crone, 2017a; Spaans *et al.*, 2018; Brandner *et al.*, 2020).

Compared with the decrease in responses observed when winning rewards for friends or charity donations, as opposed to winning for oneself, a handful of studies have reported results showing the distinctiveness of winning for family members. For example, participants showed comparable electrophysiological responses in the FRN/RewP component when winning for themselves and their family members (e.g. mother and father) in a study using the SGT (Zhu *et al.*, 2016b). Yet, less is known about the experience of mothers when they win rewards for their children. Only one neuroimaging study has reported that mothers experience the most enjoyment when they are winning rewards for their adolescent children, compared to when they are winning rewards for themselves. More activity in the social brain regions, such as the dorsomedial prefrontal cortex, precuneus and TPJ, was observed in the former (child) condition than in the latter (self) condition (Spaans *et al.*, 2018).

Neurobehaviorally, recent studies have provided evidence of alterations in rodents’ mesolimbic dopaminergic (DA) system (i.e. the neuronal pathway from the ventral tegmental area to the nucleus accumbens, amygdala and prefrontal cortex) during pregnancy and postpartum, which can promote bias toward offspring-related stimuli and give rise to motivated maternal behaviors (see review by Rincón-Cortés and Grace, 2020). The same regions in the brains of human mothers were also found to be more activated when they saw pictures of their own infants, and the activation was modulated by the individual’s attachment style (Strathearn *et al.*, 2009; Ulmer-Yaniv *et al.*, 2022). Mother–child attachment is unique and present throughout the child’s entire developmental process (see review by Brumariu and Kerns, 2010). New mothers often sacrifice their own time and energy to meet their children’s needs and even put their children’s well-being above their own needs (Horne and Breitkreuz, 2018; also see the review by Nomaguchi and Milkie, 2020). The following question is whether this particular relationship between a mother and a young child may affect the mother’s reward sensitivity rooted in the DA system and hence break the self-prioritization effect on the reward-pursuing process.

In the present study, we aimed to fill this knowledge gap by investigating the neural mechanisms of mothers when they win rewards for their young children, compared with winning them for themselves or for a charity donation. We used the MID paradigm to conduct a parallel analysis of event-related potential (ERP) and event-related spectral perturbation (ERSP) data to investigate the underlying neural activity. In each trial, a cue indicating the reward beneficiary, i.e. self (S), child (C) or donation (D), was followed by a task requiring a fast and accurate response, after which feedback indicating whether the designated beneficiary gained the reward was presented.

We evaluated the cue-P2 and contingent negative variation (CNV) during the reward anticipation phase, as well as the RewP and feedback-P3 components during the reward consumption phase. For the anticipation phase, the cue-P2 was a positive-going component (peaking at ∼200 ms post-cue onset) thought to reflect early motivational relevance and attentional allocation (Carretié *et al.*, 2001; San Martín *et al.*, 2010; Flores *et al.*, 2015). The CNV is a slow negative-going component (peaking at ∼1000 ms post-cue onset), reflecting stimulus anticipation and motor preparation of the upcoming stimulus (Walter *et al.*, 1964; Tecce, 1972; van Boxtel and Brunia, 1994; Frömer *et al.*, 2021). In the consumption phase, the RewP component (peaking between 250 and 300 ms post-feedback onset) is thought to reflect context expectation (Flores *et al.*, 2015; Zhang *et al.*, 2020) and outcome valence (Pornpattananangkul and Nusslock, 2015; Krugliakova *et al.*, 2019). Following the RewP component, the feedback-P3 component (which peaks between 300 and 400 ms post-feedback onset) is related to feedback-guided learning and outcome evaluation (Hajcak *et al.*, 2007; Pornpattananangkul and Nusslock, 2015).

We used time–frequency analysis to identify further the neural mechanisms in altruistic reward processing, given the limited literature on this topic (Glazer *et al.*, 2018; Meyer *et al.*, 2021). Previous evidence suggests that delta/theta oscillations are associated with motivational processes in both the anticipation and consumption phases (Bernat *et al.*, 2011; Nelson *et al.*, 2011; Foti *et al.*, 2015; Paul and Pourtois, 2017; Watts *et al.*, 2018; Cavanagh *et al.*, 2019). For instance, delta (1–3 Hz) and theta (4–7 Hz) oscillations are sensitive to monetary gains and losses, respectively (Cohen *et al.*, 2007; Glazer *et al.*, 2018). However, there is little evidence regarding time–frequency representations of neural activity in altruistic reward processing.

In accordance with previous studies, we expected that gaining rewards for children would elicit comparable or even higher brain responses, as reflected in the ERP and ERSP responses, compared to gaining rewards for oneself. We also anticipated that these two conditions would elicit higher neural responses compared to the donation condition. The results of recent studies (e.g. Frömer *et al.*, 2021; Rong *et al.*, 2022) and the theory of the expected value of control (Shenhav *et al.*, 2013) support the premise that the expected value of a reward determines the cognitive effort devoted to a given task. Hence, we predicted that higher cue-related ERP responses (including the cue-P2, CNV and cue-delta/theta power) would predict better task performance.

Furthermore, electroencephalogram (EEG) source localization was applied to assess ERP activity using the standardized low-resolution brain electromagnetic tomography analysis (sLORETA) during the anticipation phase. The purpose was to identify different neural representations of the motivated behavior of gaining rewards for different beneficiaries. Based on previous neuroimaging evidence (Spaans *et al.*, 2018), we predicted that the social brain regions (i.e. the TPJ and precuneus) would be more active in the child condition relative to the self and donation conditions. Moreover, the brain regions associated with attentional and cognitive control (i.e. the precentral gyrus) would be more active in the self condition compared to other conditions (Lockwood *et al.*, 2022).

## Method

### Participants

A total of 31 mothers between 20 and 45 years old [mean (*M*) = 34 years, standard deviation (s.d.) = 2.69] participated in this study. One participant was excluded from further analyses due to technical problems during the EEG recording. Two participants were excluded due to incomplete data. Hence, the final sample included 28 participants (age: *M* = 32, s.d. = 2.33). All participants had attained a bachelor-level qualification or higher and were married. Each participant had only one child aged 2–6 years at the time of participation (*M* = 4, s.d. = 1.23). All participants were right-handed and had normal or corrected-to-normal vision. None of them reported a history of neurological, psychiatric or mental disorders. The Ethics Committee of the School of Psychology at Capital Normal University approved the study and all participants gave their informed consent before participation, in accordance with the Declaration of Helsinki.

### Design and procedure

**Fig. 1.**
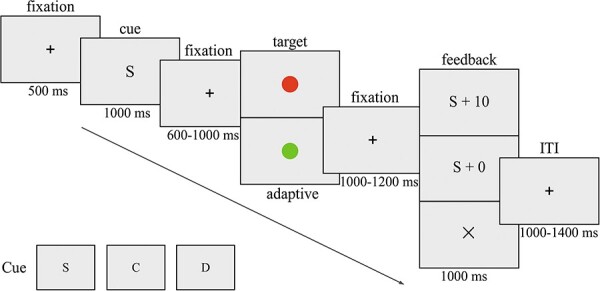
An overview of the task and trial structure. The letter ‘S’ (self) indicates the participant, ‘C’ (child) indicates the participant’s child and ‘D’ (donation) indicates a donation to a charity program with a mission to help children in need.

A within-participant factorial design with three levels of reward beneficiary (winning rewards for oneself, one’s child or a donation) was employed. Participants were seated in a dimly lit and sound-attenuated room. Presentation software (https://www.neurobs.com/) was used for the delivery of stimuli and recordings of response times (RTs) and error rates. All stimuli were presented on a black background. At the beginning of each trial (Figure 1), a white fixation cross (0.39° × 0.39° on a visual angle) appeared at the center of the screen for 500 ms, followed by a visual cue (1.15° × 1.15° on a visual angle), which was displayed in the center of the screen for 1000 ms. The cue indicated the different reward beneficiaries. Specifically, the letter ‘S’ indicated winning rewards for the self, which was the participant, ‘C’ indicated winning rewards for the participant’s child and ‘D’ indicated winning rewards for a donation to a charity program with a mission of helping children in need. A charity donation program was chosen by each participant from the Alipay app (https://www.alipay.com/) before the formal experiment. We also had a baseline no-reward condition indicated by the letter ‘N’ during the cue phase, in which participants would receive no additional monetary reward. However, the no-reward condition was omitted from the later analyses for simplicity and clarity because we focused on comparisons among the three current conditions (S, C and D) in the present study. After a variable cue-target interval (CTI) of 600–1000 ms, the target (1.23° × 1.23° on a visual angle) was presented at the center of the screen. The use of variable CTIs can prevent participants from forming time-based expectations for the target’s onset. Therefore, target stimuli were randomly presented at the center of the screen as a circle (diameter: 1.5 cm) and were colored either red (RGB: 215, 44 and 17) or green (RGB: 155, 251 and 68). Participants were required to judge the color of the target circle as fast and as accurately as possible by pressing the ‘F’ or ‘J’ button with their index fingers of their left and right hands. The response keys were counterbalanced between participants. The duration of the target was varied by a step of 25 ms per condition, with a duration of 300–750 ms. This adjustment ensured that participants’ hit rate would be close to 50% in each reward beneficiary condition, and participants would receive similar amounts of positive and negative feedback messages, sufficient for the EEG analysis. Specifically, if a participant responded correctly and faster than their baseline RT (the initial baseline RT was determined by the practice session, see later) in the current reward beneficiary condition, the baseline RT would decrease by 25 ms in the following trial with the same reward beneficiary condition. In contrast, if a participant responded correctly but slower than the baseline RT in the current reward beneficiary condition, the baseline RT would increase by 25 ms in the following trial of the same reward beneficiary condition. After participants responded or the target disappeared, the fixation cross was presented again for 1000–1200 ms. Subsequent trial feedback in the form of ‘condition type + 10/+ 0’ (e.g. ‘S + 10’ and ‘D + 0’) appeared for 1000 ms in the center of the screen to inform the participant of their performance in the current trial. The positive feedback of ‘+10’ was presented following responses that were correct and faster than the baseline RT, whereas the negative feedback of ‘+0’ was presented following responses that were correct but slower than the baseline RT. The feedback of ‘×’ was presented following incorrect responses. Upon termination of the feedback stimulus, the fixation cross was presented for 1000–1400 ms (inter-trial interval).

The formal task consisted of 480 trials, presented over 10 blocks (with 12 trials for each reward beneficiary condition per block), and all types of trials were presented in a pseudo-random order. After each block, a short break was included. Participants were required to practice 48 trials before the formal experiment. During the practice session, participants were informed that the cue letters were task-irrelevant and they were instructed to ignore them. They were required to discriminate the color of the target circle as quickly and accurately as possible by pressing the ‘F’ or ‘J’ button. Participants only received ‘correct (√)’ and ‘false (×)’ feedback during the practice session. The averaged correct RT for each participant during the practice session was used as the participant’s baseline RT in the first trial of each reward beneficiary condition.

After the practice session, participants were informed of the meanings of the cue letters and the presentation rule for the feedback in the formal experiment. To ensure that participants understood the correct meanings of the cue letters, they were asked to complete a test before the formal experiment. Specifically, the meanings of the cues were randomly selected and presented at the center of a screen (e.g. self/child/donation in Chinese), and participants were required to press the button corresponding to the cue letter on the computer keyboard (i.e. S/C/D buttons). The formal experiment began after judgments regarding all three conditions were correct. Prior to the formal experiment, we informed participants that in addition to the base pay of 200 Chinese Yuan for the completion of the experiment, they could gain extra rewards depending on their task performance. After the experiment, the final reward points for each condition would transform into extra rewards for themselves, their children and the program they selected for a donation. Due to the adaptive algorithm of the task, the participants received similar numbers of positive and negative feedback messages. The extra rewards for themselves (a box of hand cream) and for their child (a set of jigsaw puzzles) were given to the participant after the experiment (participants did not know what the gift was until they had finished the experiment). For the donation condition, we calculated each positive feedback as 0.3 Chinese Yuan and donated it to the charity program that was chosen by the participant in the presence of a witness. The value of the extra reward was ∼20 Chinese Yuan for each condition.

### Self-report questionnaires

After the formal experiment, participants answered several questions pertaining to themselves, their children and the donation while performing the MID task: (i) how pleasant they felt when they won a reward for themselves, their children and the charity donation; (ii) how much they thought themselves, their children and the charity program deserved to win a reward; and (iii) how hard they worked to win a reward for themselves, their children and the charity donation. All questions were answered on a 7-point scale ranging from 1 = not at all to 7 = very much.

To assess individual differences in the inhibition and approach tendencies toward rewards, participants were asked to complete the Behavioral Inhibition System (BIS)– Behavioral Activation System (BAS) scale (Carver and White, 1994). In order to measure the closeness between the mother and child, participants completed the Inclusion of Other in the Self (IOS) scale (Aron *et al.*, 1992), which was developed as a Venn diagram. The IOS scale has been evaluated to be a meaningful and reliable measure of the subjective closeness of relationships (Gächter *et al.*, 2015). The measure depicts two circles: one circle represents the ‘self’ (i.e. the participant) and the other circle represents the ‘other’ (i.e. the participant’s child). Seven depictions of the Venn diagram were provided, beginning from no overlap of the circles (score 1) to overlapping each other increasingly (scores 2–7). Participants received the following instructions: ‘Please select the one which best reflects the relationship between you and your child. The more the circles intersect, the closer the relationship is.’ We also used the Self-Report Altruism Scale Distinguished by the Recipient (SRAS-DR) to measure altruistic tendencies toward different recipients, including family members, friends and strangers (Oda *et al.*, 2013).

### Electrophysiology recordings and pre-processing

Electroencephalogram (EEG) recordings were obtained from 64 scalp sites using Ag/AgCl electrodes embedded in an elastic cap at locations derived from the extended International 10–20 system (NeuroScan; Compumedics, EI Paso, TX, USA). These electrodes were referenced to the right mastoid during recording. Vertical and horizontal electro-oculograms were recorded with two pairs of bipolar montages: one pair of electrodes was placed above and below the left eye, and the other pair was placed 10 mm from the lateral canthi. The impedance of all electrodes was reduced <5 KΩ and the sample rate was 500 Hz online. All EEG signals were pre-processed offline with EEGLAB (www.sccn.ucsd.edu/eeglab), an interactive MATLAB toolbox (R2018b). The EEG signals were referenced to the average of the right and left mastoid potentials (the M1 and M2 electrodes) and were filtered with a band-pass of 0.5–30 Hz. Artifacts, including eye movements and blinks, were rejected using independent component analysis. The averages of the epochs for the reward anticipation and the consumption phases were 1600 and 2000 ms, respectively, with an additional 200 ms recorded before stimulus onset as a baseline in the time domain. Baseline corrections were performed using the mean amplitude of the pre-stimulus onset. Trials with a voltage, relative to the 200 ms baseline, exceeding ±85 μV at any electrode were excluded from the analysis. The average percentages of the excluded trials in the cue and feedback phases were 7.34% and 8.83%, respectively, resulting in 105 valid trials per cue condition and 52 valid trials per feedback condition (positive *vs* negative in each reward beneficiary condition) on average.

### Data acquisition and analysis

#### Behavioral data analysis

We excluded RTs from the analysis (1.56% of total trials) that were >3 s.d above or below the mean in each experimental condition for each participant. The trial-by-trial RTs, accuracy (correct *vs* incorrect) and feedback (positive *vs* negative and incorrect feedback) for each reward beneficiary condition were analyzed.

#### Time-domain analysis of EEG data

We computed ERP responses for the cue-locked P2 (cue-P2, 140–225 ms, averaged across Fz, FC1, FCz, FC2 and Cz), CNV (cue-CNV, 1300–1600 ms, averaged across Fz, FC1, FCz, FC2 and Cz), RewP (200–280 ms, averaged across FCz, C1, Cz, C2 and CPz) and P3 (feedback-P3, averaged across FCz, C1, Cz, C2 and CPz). Please note that due to the feedback-P3 latency between the positive and negative feedback being different, we calculated single-trial feedback-P3 amplitudes by extracting a 200 ms window centered around the peak for each trial between 280 and 500 ms post-feedback onset.

#### Time–frequency analysis of EEG data

After all EEG data were pre-processed, an estimation of the oscillatory power as a function of time and frequency (time–frequency representation) was obtained from single-trial EEG epochs using a short-time Fourier transform (STFT) with a fixed 400 ms Hanning-tapered window. Time–frequency representations were explored from 1 to 10 Hz in steps of 0.5 Hz. For each epoch, the STFT algorithm yielded a complex time–frequency estimate F(*t*, *f*) at each time–frequency point (*t*, *f*), which extended from −600 to 1600 ms (in steps of 1 ms) in the cue phase and −600 to 2000 ms (in steps of 1 ms) during the feedback phase. The spectrograms were baseline-corrected with a reference interval ranging from −400 to −100 ms for both the cue phase and the feedback phase (i.e. by subtracting the average power of the pre-stimulus interval from the power at each post-stimulus time point) for each trial.

Time–frequency data were analyzed in the lower frequency band (<10 Hz), which has been reported to be involved in reward processing (Cohen *et al.*, 2007; Cavanagh, 2015; Jin *et al.*, 2019). The regions of interest were defined to extract the magnitude of the time–frequency distribution at fronto-central regions of the delta/theta band (Fz, FC1, FCz, FC2 and Cz). Statistical analyses were conducted on the cue-delta/theta-event-related synchronization (ERS) with average power values from 100 to 350 ms post-cue onset in 1–8 Hz and the feedback delta ERS with an average power ranging from 200 to 500 ms post-feedback onset in 1–5 Hz.

#### Source analysis (sLORETA)

After all EEG data were pre-processed, average referencing was performed before further analysis. We used the sLORETA software (v20081104, https://www.uzh.ch/keyinst/loretaOldy.htm, Pascual-Marqui, 2002) to estimate the neural sources underlying comparisons between the activation patterns of the different reward beneficiary conditions (i.e. self *vs* child, self *vs* donation and child *vs* donation). The sLORETA software computes the current density at the source of the signal without assuming a pre-defined number of sources. The software used in the MNI152 template produced a total of 6239 cortical gray matter voxels at 5 mm resolutions (Lancaster *et al.*, 2000).

Electrode coordinates were generated from the 60 electrode locations. A transformation matrix was created using electrode coordinates. The averaged waveforms (from 0 to 1600 ms post-cue onset) for each participant were converted and saved to a two-dimensional matrix including electrodes and ERP amplitudes. Source localization consisted of ERP and sLORETA analyses. During the ERP analysis, we focused on the time window for cue-P2 (140–225 ms) based on the results of the time domain. The source-localized amplitude was averaged across the time points that showed a significant difference in the ERP analysis, which was calculated using point-by-point paired *t*-tests for each electrode to determine the significant period. The test was subject to permutation testing using 5000 randomizations, and it was corrected for multiple comparisons using the Bonferroni correction. The *P*-value of the test in which the maximum occurred <0.05 was considered the threshold for deciding the significant region of interest of time samples of activity. During the sLORETA analysis, the mean amplitude of the groups was compared using multiple voxel-by-voxel comparisons and paired group *t*-tests (i.e. self *vs* child, self *vs* donation and child *vs* donation). The tests were also subject to permutation testing using 5000 randomizations, and they were corrected for multiple comparisons using the Bonferroni correction. The sLORETA values for each participant were computed using ASCII values, electrode coordinates and a transformation matrix, separately. The results were evaluated for their levels of significance, and their values were displayed with a three-dimensional brain model.

### Statistical analysis

Statistical analyses and plots were performed using MATLAB (R2018b) and R4.1.2 (R Core Team, 2020). All pre-processed trials were analyzed using linear mixed-effects models (LMMs) or generalized LMMs (GLMMs; Meteyard and Davies, 2020).

For the behavioral data, an LMM was conducted on the correct RT for each trial, and GLMMs on the accuracy (correct *vs* wrong) and feedback (positive *vs* negative feedback) per trial. For the EEG data, the cue-locked EEG (ERPs and ERSPs) models for each earlier index (i.e. the cue-P2 and cue-delta/theta ERS) were analyzed separately with reward beneficiary as a fixed factor; those for each later index (i.e. CNV) were extended by including the neighboring earlier index as another fixed factor to control for the influence of the former. The same logistic models were used for the feedback-locked EEG data (i.e. RewP and feedback delta ERS for the earlier index and feedback-P3 for the later index). This analysis was conducted with feedback valence (positive and negative) and the interaction between reward beneficiary and feedback valence added as predictors to examine the neural activity of the gain and no-gain rewards and the influence of the beneficiary on both. Participants were included in all of the models as a random factor to control for the variance across participants. Moreover, a one-way repeated-measures analysis of variance was performed to examine separately the results of the three self-report scales (pleasantness, deservedness and effort scores) with the reward beneficiaries (S, C *vs* D) as a within-participant factor. We calculated only basic descriptive statistics (*M* and s.d.) for the BIS–BAS, IOS and SRAS-DR scales.

To investigate the overall effects of EEG activity on behavioral performance (i.e. RT), LMMs of RTs were performed separately on single-trial cue-locked ERPs (CNV and cue-P2) and ERSPs (cue-delta/theta ERS) with reward beneficiary, cue-locked ERPs/ERSPs, and all their interactions as predictors, and individual variation as a random factor. The LMM and GLMM analyses were conducted using the lmer program of the lme4 CRAN package (http://CRAN.R-project.org/package=lme4). The significance of the fixed effect was analyzed using the lmerTest package (http://CRAN.R-project.org/package=lmerTest). *Post hoc* tests were analyzed using the emmeans package (http://CRAN.R-project.org/package=emmeans); the significance level was set to 0.05 and either Bonferroni-pairwise or simple main-effects comparisons were conducted where appropriate.

## Results

### Behavioral performance

As expected, the results of the LMM analysis of RTs revealed a main effect of reward beneficiary, *F*(2, 9425) = 105.94, *P* < 0.001. *Post hoc* pairwise analyses revealed that the RTs in the child condition were significantly shorter than those in the self (*b* = −7.72, *z* = −3.31, *P* = 0.003) and donation (*b* = −32.55, *z* = −13.92, *P* < 0.001) conditions, and the RTs in the self condition were significantly shorter than those in the donation condition (*b* = −24.83, *z* = −10.64, *P* < 0.001).

The results of the LMM analyses of accuracy and feedback revealed no significant effect of reward beneficiary (accuracy: χ^2^ = 1.17, *P* > 0.5; feedback: χ^2^ = 1.01, *P* > 0.5), primarily due to the adaptive algorithms during the target phase.

### Cue-locked EEG results

#### Cue-P2

The LMM results revealed a main effect of reward beneficiary, *F*(2, 8817.1) = 16.91, *P* < 0.001. Compared with the donation condition, the self and child conditions elicited more positive-going cue-P2 responses (S > D: *b* = 0.58, *z* = 2.71, *P* = 0.020; C > D: *b* = 1.24, *z* = 5.81, *P* < 0.001). The child condition also elicited more positive-going ERP responses than the self condition (C > S: *b* = 0.66, *z* = 3.09, *P* = 0.006).

#### Cue-CNV

**Fig. 2.**
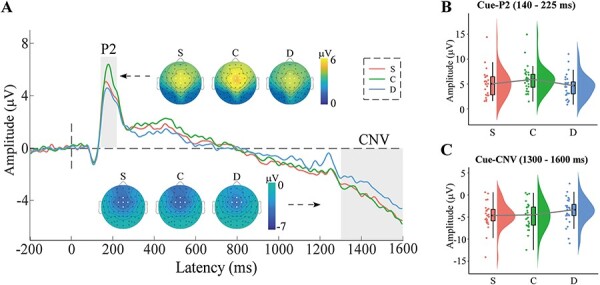
Cue-locked time-domain results. (A) The grand average ERP waveforms including the cue-P2 (140–225 ms) and CNV (1300–1600 ms) components across five electrodes (Fz, FC1, FCz, FC2 and Cz) and the topographic maps for the three reward anticipation conditions (S, C and D). (B, C) Raincloud plots of the mean amplitudes of the cue-P2 and CNV components. S = winning rewards for oneself, C = winning rewards for one’s child and D = winning rewards for donation to a charity program.

The LMM results revealed a significant main effect of reward beneficiary, *F*(2, 8817.1) = 27.95, *P* < 0.001, with more negative-going CNV waveforms for the self and child conditions, compared to the donation condition (S < D: *b* = −1.12, *z* = −5.07, *P* < 0.001; C < D: *b* = −1.61, *z* = −7.30, *P* < 0.001), whereas the amplitudes of the self and child conditions were comparable (*b* = 0.49, *z* = 2.23, *P* > 0.05). The fixed effect of the cue-P2 on CNV was significant, *F*(1, 8840.9) = 1127.36, *P* < 0.001, indicating that a more positive cue-P2 amplitude was associated with a more positive (less negative) CNV amplitude (*b* = 0.37). Figure 2 depicts the cue-locked time-domain results.

#### Cue-delta/theta ERS

**Fig. 3.**
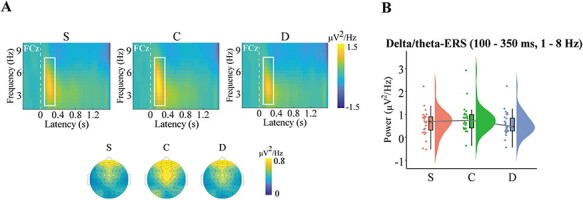
Cue-locked time–frequency results. (A) Oscillatory responses in delta/theta-ERS (100–350 ms, 1–8 Hz) oscillations across five electrodes (Fz, FC1, FCz, FC2 and Cz). (B) Raincloud plots of the power of the delta/theta-ERS component for each condition. S = winning rewards for oneself, C = winning rewards for a child and D = winning rewards for a charity donation.

The LMM results revealed a significant effect of reward beneficiary, *F*(2, 8743.3) = 7.79, *P* < 0.001. The power in the child condition was significantly stronger than that of the self and donation conditions (C > D: *b* = 0.23, *z* = 3.78, *P* < 0.001; C > S: *b* = 0.17, *z* = 2.86, *P* = 0.013), whereas the power of the self and donation conditions were comparable (*b* = 0.06, *z* = 0.92, *P* > 0.05, Figure 3).

### Feedback-locked EEG results

**Fig. 4.**
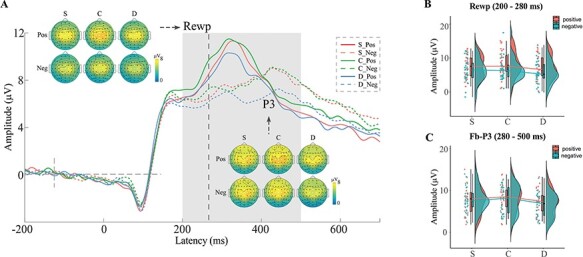
Feedback-locked time-domain results. (A) The grand average ERP waveforms of RewP (200–280 ms) and feedback-P3 (200 ms window centered around the peak which ranged in 280–500 ms for each trial) components across five electrodes (FCz, C1, Cz, C2 and CPz) and the topographic maps in the feedback phase. (B, C) Raincloud plots of the mean amplitudes of RewP and feedback-P3 components for each condition. S = winning rewards for self, C = winning rewards for one’s child and D = winning rewards for donation to a charity program.

Figure 4 shows the feedback-locked time-domain results. The analysis of the RewP revealed the main effect of reward beneficiary was significant, *F*(2, 8359.2) = 6.32, *P* = 0.002. Compared with the donation condition, the feedback in the child condition elicited more positive-going RewP responses (C > D: *b* = 0.81, *z* = −3.54, *P* = 0.001), whereas no significant differences were found in the other comparisons (D *vs* S: *b* = −0.33, *z* = −1.46, *P* > 0.05; S *vs* C: *b* = −0.48, *z* = −2.07, *P* > 0.05). The main effect of feedback valence was significant, *F*(1, 8360) = 54.73, *P* < 0.001, with more positive-going RewP responses elicited for positive feedback compared to negative feedback (*b* = 1.38, *z* = 7.40, *P* < 0.001). The interaction between reward condition and feedback valence was not significant, *F*(2, 8359.4) = 0.40, *P* > 0.05.

The feedback-P3 latency was significantly earlier in positive feedback compared with that in negative feedback (*P* < 0.001, 390 *vs* 420 ms). Thus, we calculated feedback-P3 by extracting single-trial feedback-P3 amplitudes (see ‘Method’ section). The results showed that the main effect of reward beneficiary was significant, *F*(2, 8359.3) = 9.71, *P* < 0.001. The feedback in the self and child conditions elicited more positive-going feedback-P3 responses than that in the donation condition (S > D: *b* = 0.70, *z* = 3.92, *P* < 0.001; C > D: *b* = 0.66, *z* = 3.70, *P* < 0.001), whereas the difference in the amplitudes between the self and child conditions was not significant (*b* = 0.04, *z *= 0.22, *P* > 0.05). The main effect of feedback valence, *F*(1, 8360.5) = 0.43, *P* > 0.05, and the interaction effect between reward beneficiary and feedback, *F*(2, 8359.5) = 1.27, *P* > 0.05, were not significant. Moreover, the fixed effect of RewP on the feedback-P3 was significant, *F*(1, 8386.8) = 6652.25, *P* < 0.001, indicating that a more positive RewP amplitude was associated with a more positive feedback-P3 amplitude (*b* = 0.69, Figure 4). No significant effects were found in the analysis of the feedback-related delta ERS (*P* > 0.05).

### The effect of time-domain ERP activity on RTs

**Fig. 5.**
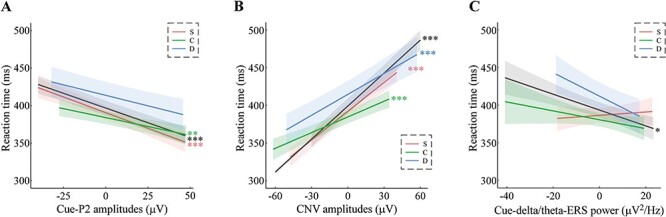
Effects of time-domain and time–frequency activity on RTs. (A, B) The fixed effects of time-domain analysis on RTs for the amplitude of the cue-P2 and CNV by the three reward beneficiaries (S, C and D). (C) The fixed effects of the time–frequency analysis on RTs for the power of the delta ERS by the three reward beneficiaries (S, C and D). The black lines indicate the main fixed effects of the cue-P2 (A), CNV (B) and cue-delta/theta ERS (C) on RTs, and the shaded areas represent the 95% confidence intervals. S = winning rewards for oneself, C = winning rewards for one’s child and D = winning rewards for a charity donation; * *P* <0.05, ** *P* < 0.01, *** *P* < 0.001 after the Bonferroni correction.

To investigate how the EEG responses during the cue phase affected RTs, analyses of the RTs using LMMs were performed to examine the time-domain EEG responses during the cue phase. The time-domain results of the RTs revealed a main effect of reward beneficiary on RTs, *F*(2, 8817.8) = 45.62, *P* < 0.001, showing the same pattern as the results of the LMM analysis of RTs (C < S < D, see earlier). The main effect of the cue-P2 was significant, *F*(1, 8837.6) = 25.32, *P* < 0.001, suggesting that a more positive cue-P2 amplitude was associated with a faster RT (Figure 5A). The main effect of the CNV was significant, *F*(1, 8837.5) = 102.32, *P* < 0.001, suggesting that a more negative CNV amplitude was associated with a faster RT (Figure 5B). The CNV’s predictive effect on RT was significantly stronger than that of the cue-P2 (χ^2^ = 14.53, *P* < 0.001), thereby providing evidence that the cue-locked CNV may play a more important role in shaping RTs during the target phase. Other effects were not significant, *P* > 0.05.

The results of the effects of the time–frequency domain responses on participants’ RTs revealed a main effect of reward beneficiary, *F*(2, 8743.1) = 83.49, *P* < 0.001, replicating the LMM results reported earlier. The main effect of the delta/theta ERS was significant, *F*(1, 8751.2) = 4.90, *P* = 0.027, indicating that a more powerful delta/theta ERS was associated with faster RTs (Figure 5C). Although the interaction between reward beneficiary and the delta/theta ERS was significant, *F*(2, 8743.8) = 3.45, *P* = 0.032, the LMM results for each reward condition with the delta/theta ERS as a predictor revealed no significant fixed effects of the delta/theta ERS in the self [*F*(1, 2899) = 0.10, *P* > 0.5], child [*F*(1, 2917.7) = 0.75, *P* > 0.1] or donation condition [*F*(1, 2891.3) = 3.08, *P* > 0.05].

### sLORETA analysis

**Fig. 6.**
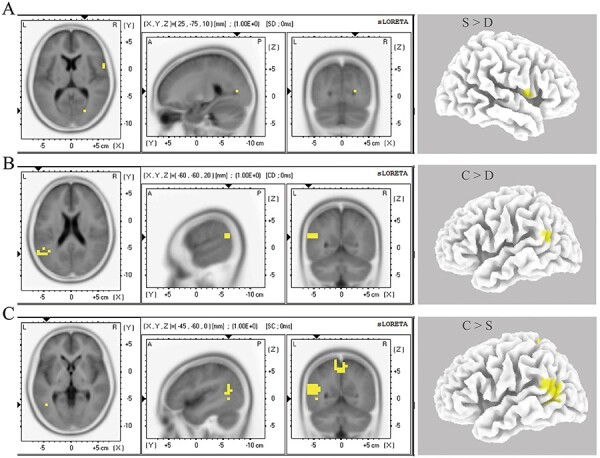
sLORETA results during the cue phase. (A) Stronger activity in the precentral gyrus and cuneus was revealed in the self condition than in the donation condition. (B) Stronger activity in the supra-marginal gyrus and superior temporal gyrus was observed in the child condition than in the donation condition. (C) Stronger activity in the temporoparietal junction, precuneus and post-central gyrus was observed in the child condition than in the self condition. The highlighted areas in the images indicates significant positive *t*-values. Only significant voxels (*P* < 0.05) are reported. S = winning rewards for oneself, C = winning rewards for one’s child and D = winning rewards for donation to a charity.

The source localization analysis revealed that at latencies ranging from 156 to 170 ms, stronger activity was present in the self, relative to the donation condition, localized in the precentral gyrus [the Brodmann area (BA): 44, Montreal Neurological Institute (MNI): *x* = 60, *y* = 5, *z* = 10, *P* < 0.05], and in the cuneus [BA 30, MNI: *x* = 25, *y* = −70, *z* = 10, *P* < 0.05, Figure 6A].

At latencies ranging from 204 to 224 ms, the analysis revealed stronger activity in the child condition relative to the donation condition, localized in the supra-marginal gyrus (BA 40, MNI: *x* = −60, *y* = −55, *z* = 20, *P* < 0.05) and the superior temporal gyrus (BA 39, MNI: *x* = −55, *y* = −60, *z* = 20, *P* < 0.05, Figure 6B).

At latencies ranging from 164 to 198 ms, the analysis revealed stronger activity in the child condition relative to that in the self condition, localized in the middle temporal gyrus (BA 37, MNI: *x* = −50, *y* = −65, *z* = 5, *P* < 0.05), the precuneus (BA 7, MNI: *x* = −5, *y* = −50, *z* = 55, *P* < 0.05) and the post-central gyrus (BA 5, MNI: *x* = 5, *y* = −45, *z* = 65, *P* < 0.05, Figure 6C).

### Questionnaire rating results

Behavioral ratings revealed that the main effect of the self-reported pleasantness scores was significant, *F*(2, 54) = 8.054, *P* < 0.001, $\eta _{p{\ }}^2$ = 0.230. Compared with the donation condition, the subjective pleasantness of winning rewards in the child condition was more pronounced (C > D, 6.7 > 6.1, *t *= 4.076, *P *= 0.001, Cohen’s *d* = 0.770), whereas no significant differences were found in the other comparisons (S *vs* D, 6.4 *vs* 6.1: *t *= 1.760, *P *= 0.269, Cohen’s *d* = 0.333; S *vs* C, 6.4 *vs* 6.7: *t *= 2.202, *P *= 0.109, Cohen’s *d* = 0.416). Moreover, the main effect of the self-reported effort scores was significant, *F*(2, 54) = 6.435, *P* = 0.003, $\eta _{p{\ }}^2$ = 0.192. Compared with the self and donation conditions, the subjective effort scores for winning rewards in the child condition were more pronounced (C > S, 6.8 *> *6.5: *t *= 3.286, *P *= 0.008, Cohen’s *d* = 0.621; C > D, 6.8 > 6.4: *t *= 3.306, *P *= 0.008, Cohen’s *d* = 0.625), whereas no significant differences were found between the self and donation conditions (S *vs* D, 6.5 *vs* 6.4: *t *= 0.827, *P *= 1.000, Cohen’s *d* = 0.156). The main effect of the self-reported deservedness scores was not significant *F*(2, 54) = 1.246, *P* = 0.289, $\eta _{p{\ }}^2$ = 0.044. Descriptive statistics for the results of the questionnaire are presented in Table 1.

**Table 1.** Descriptive statistics of the data from the three self-report questionnaires

Scales	Min.	Max.	*M*	s.d.
BIS	10	20	15.2	2.6
BAS-Drive	5	11	8.7	1.7
BAS-Fun Seeking	4	14	9.4	1.9
BAS-Reward Responsiveness	5	13	8.7	1.9
BAS Total	14	38	26.8	5.5
IOS	3	7	5.9	1.2
SRAS-Family Members	27	35	32.0	2.6
SRAS-Friends	24	35	30.5	3.3
SRAS-Strangers	19	35	27.1	4.2
SRAS Total	75	105	89.6	8.8

## Discussion

The goal of the current study was to examine the possible distinctiveness of mothers’ neural responses when gaining rewards for their children. For this purpose, we compared the behavioral responses and the temporal neural dynamics of reward processing in the MID task in which mothers won rewards for themselves, their children and charity donations. As expected, the behavioral results revealed significantly shorter RTs in gaining rewards for children compared with those in gaining rewards for themselves or donations, and shorter RTs in gaining rewards for themselves than those in gaining rewards for donations (C < S < D). These behavioral results indicate that participants exerted the greatest effort when obtaining rewards for their children, followed by themselves, and exerted the least effort when obtaining rewards for donations, which is consistent with their self-reports that they experienced more pleasure and exerted greater effort when winning rewards for their children, compared to when winning rewards for those in the other conditions.

In order to study the neural mechanisms of reward processing in different reward beneficiary conditions, we analyzed the time-domain and time–frequency domain signals in the reward anticipation and reward consumption phases, respectively. Consistent with the pattern of the behavioral results, we found that the amplitude of the cue-P2 component was significantly influenced by reward beneficiary during the anticipation stage, with the strongest responses in the children’s condition (C > S > D). These results most likely reflect early bottom-up attentional engagement associated with the DA reward system and motivational salience (Potts, 2004; Potts *et al.*, 2006; but see Luo *et al.*, 2019). Evidence from research across animals and humans has demonstrated that pregnancy and postpartum bring about the brain’s structural and functional adaptive changes for maternal motivation, thereby enabling the survival and development of the offspring (Kim *et al.*, 2010; Hoekzema *et al.*, 2017; Barba-Müller *et al.*, 2019). Neuroimaging studies have reported that reward circuitry plays a crucial role in sensitive maternal behavior, especially between a mother and her young child. For example, the reward circuit in the mother’s brain is sensitive to infant-related cues (Strathearn *et al.*, 2009), and the reward-related regions (including the ventral tegmental area, substantia nigra and ventral striatum) were activated selectively when mothers viewed their own infant’s face, compared to the face of an unknown infant (Nitschke *et al.*, 2004; Noriuchi *et al.*, 2008; Strathearn *et al.*, 2008; Strathearn and Kim, 2013; Kluczniok *et al.*, 2017). The current P2 results suggest that early enhancement induced by a child’s cue may reflect a more important signal of incentive salience, which elicited stronger reward-related neural responses to prepare for exerting the effort necessary to win the rewards, compared with winning rewards for those in the other conditions.

Similar to the cue-P2 component, we also found that the power of the cue-delta/theta ERS was significantly stronger in the child condition, compared to the self and donation conditions. Increased delta and theta power values have been assumed to reflect enhanced top-down control over behavior (Cavanagh *et al.*, 2013, 2014), stronger motivational salience (see reviews: Knyazev, 2007, 2012) and greater mental effort (Gevins *et al.*, 1998; Jensen and Tesche, 2002; Shaw *et al.*, 2018; Yu *et al.*, 2022). Therefore, this pattern of results is consistent with our hypothesis and supports the statement that the mothers exerted greater attentional control and effort to win rewards for their children than they did to win rewards for themselves and donations during the reward anticipation stage.

Although the early P2 component revealed a child-specific pattern, we observed comparable CNV amplitudes for the child and self conditions that were larger for these two conditions compared to the donation condition in the later time window. Considering that CNV reflects the proactive control and motor preparation for the upcoming target (Schevernels *et al.*, 2014; Glazer *et al.*, 2018; Ait Oumeziane *et al.*, 2019), these results indicate that participants were well prepared to seek rewards for themselves and their children, rather than for a charity donation. We analyzed data to determine how neural responses in the time domain and time–frequency domain during the reward anticipation stage predicted behavioral performance. As expected, we observed that both time-domain components (cue-P2 and CNV) and time–frequency domain components (cue-delta/theta-ERS power) were significant predictors of RTs. Specifically, a more positive cue-P2 and a more negative CNV amplitude were associated with shorter RTs. Importantly, our results revealed that the CNV’s predictive effect on the RT was significantly stronger than that of the cue-P2. This finding is consistent with the notion that the P2 indexes the initial evaluation of the motivational relevance of cued incentives, but the CNV indexes the allocation and/or the execution of control over the upcoming target (Schevernels *et al.*, 2014; Cudo *et al.*, 2018; Frömer *et al.*, 2021). Furthermore, the predicted effect of the cue-delta/theta-ERS power on RTs is consistent with our recent results (Rong *et al.*, 2022). Overall, these findings support the predictions of the expected value of control theory (Shenhav *et al.*, 2013, 2017) that participants execute greater cognitive control and effort when they expect a larger reward value (i.e. winning rewards for the child in the present study).

During the consumption stage, we observed that all reward conditions elicited RewP and feedback-P3 effects. Positive feedback elicited more positive-going ERP responses than did negative feedback on RewP, which is consistent with previous ERP studies (Leng and Zhou, 2010; San Martín *et al.*, 2016; Zhu *et al.*, 2016a; Luo *et al.*, 2019; Tan *et al.*, 2022). Consistent with our hypothesis and the subjective pleasantness rating that winning rewards for the child was greater than winning them for donations, we found that winning rewards for children elicited more positive-going RewP and feedback-P3 responses than in the charity donation condition, whereas no significant differences were found for other comparisons. Prior findings have demonstrated that FRN/RewP are involved in motivational detection (Angus *et al.*, 2015; Proudfit, 2015), affective liking (Mushtaq *et al.*, 2016; Brown *et al.*, 2022) and outcome anticipation (Holroyd and Coles, 2002; Broyd *et al.*, 2012). For instance, Angus *et al*. reported that FRN/RewP amplitudes were enhanced when a person experienced approach motivation (Angus *et al.*, 2015). Mothers seemed to be significantly more motivated to win rewards for their children and experience more pleasantness than they were to win for charity donations. However, no significant difference in the RewP responses between self and charity donation was found, which is inconsistent with recent studies (Luo *et al.*, 2019; Tan *et al.*, 2022). We speculate that the mothers’ stronger concern for their child’s outcome may have degraded the reward sensitivity to their own outcome, rendering the comparison between the self and donation conditions less significant at this early stage. However, the comparison between the self and donation conditions was significant in the later feedback-P3 window, consistent with a recent observation that winning rewards for oneself elicited more positive-going ERP responses compared with winning for charity donation (San Martín *et al.*, 2016; Luo *et al.*, 2019). Many previous studies have claimed that feedback-P3 reflects attentional and motivational salience in the feedback stage (Hajcak *et al.*, 2007; Leng and Zhou, 2014; Luo *et al.*, 2019; Kwak *et al.*, 2020; Tang *et al.*, 2021). Taken together, our ERP and ERSP results provide consistent evidence that mothers show higher reward sensitivity in winning rewards for their children than they show for themselves or a charity donation.

The above-mentioned discussion is based on the main effects of the RewP and feedback-P3. The overall responses were more positive for the child condition than for the other two conditions, but they were not based on the interaction between feedback valence and reward beneficiary. The significance of the FRN/RewP was assumed to lie in differences between the positive and negative feedback (i.e. delta FRN/RewP), but this delta FRN/RewP was not modulated by reward beneficiaries in the current results. This finding was consistent with some previous studies (Kwak *et al.*, 2020), but not other studies (Leng and Zhou, 2010; San Martín *et al.*, 2016). For example, studies using the SGT have reported that the loss-minus-win FRN difference was significantly larger in the self condition than it was in a friend or charity condition, suggesting that humans can instinctively distinguish their own outcomes from those of others in the early stage of an outcome evaluation (Leng and Zhou, 2010; San Martín *et al.*, 2016). The inconsistencies in the results may be explained by variations in the experimental manipulations. The participants in the study by Leng and Zhou (2010) were not winning rewards for others (the way participants in the current study did) but were observing others’ outcomes, which may have rendered the outcomes of others as less significant than when rewards were gained by participants. In the San Martín *et al.* (2016) study, participants were required to select among four stimuli, and the selection would result in monetary outcomes for both the participant and the charity donation, with different combinations of reward valences and values, thereby introducing a more complex computation between self-gain and other-gain. Future studies are needed to further explore the variations of FRN/RewP in altruistic reward processing.

Moreover, no significant difference between the positive and negative feedback was found for feedback-P3 when controlling for the influence of the RewP on feedback-P3. This finding is inconsistent with prior ERP studies showing the feedback valence effect of feedback-P3 without controlling for the effect of RewP in conventional ERP analyses (Leng and Zhou, 2014; Luo *et al.*, 2019; Tan *et al.*, 2022). This finding may be explained by the traditional way of testing ERP components, which is to select time windows for each component and test them separately with independent analyses of variance. This method may inflate type I errors, especially when the to-be-tested two or more components are close to each other in time windows, and the later ERP component is influenced by the neighboring earlier ERP component. The use of LMMs in the present study allowed us to tease apart the effects of experimental factors and the effects of the earlier component on the later component more effectively (Bates *et al.*, 2015; Frömer *et al.*, 2021; Rong *et al.*, 2022).

Furthermore, we conducted source localization analyses to enhance our understanding of the reward processing between the self, child and donation conditions during the anticipation stage. Previous research shows that brain regions including the TPJ, precuneus and mPFC play a critical role in encoding altruistic choice and mentalizing (Van Overwalle, 2009; Murray *et al.*, 2015) and that the TPJ is a ‘boundary’ in the balancing of self–other representations (Quesque and Brass, 2019). Specifically, these brain regions exhibit increased activity when participants interact with close relatives (friends and families) than with less familiar peers (Güroǧlu *et al.*, 2008; Denny *et al.*, 2012; Spaans *et al.*, 2018; Schreuders *et al.*, 2019; Brandner *et al.*, 2020). Consistent with our hypothesis, the current results showed that the child’s cue was associated with more activity in the TPJ, compared to the self and donation conditions, suggesting that mothers recruited more social brain regions when they expected to win rewards for their children rather than for themselves or a charity donation. A recent neuroimaging study using the SGT also reported more activity in the social brain network (precuneus, dorsomedial prefrontal cortex and TPJ) when participants learned of the outcomes for their adolescent children compared to when they received rewards for themselves (Spaans *et al.*, 2018). These brain regions have also been found to be related to the empathy processing (Güroglu *et al.*, 2014; Kluczniok *et al.*, 2017; Morawetz *et al.*, 2021), suggesting that mothers may experience higher levels of shared joyfulness when they expect to win rewards for their children than when they expect to win rewards for themselves or for a charity donation. Moreover, compared with winning a reward for a donation, expecting a reward for oneself was associated with more activity in the precentral gyrus and cuneus gyrus, suggesting stronger attentional and cognitive engagement in the latter than in the former condition (Choi *et al.*, 2013; Cho *et al.*, 2016; Schreuders *et al.*, 2018; Spaans *et al.*, 2020).

The limitations of the current study should be acknowledged. First, the present design neither included other caregiving groups (e.g. fathers) for comparison, nor a control group (e.g. non-parents) for determining how unique the responses of the mothers in the child condition were. Second, our sample of participants was quite limited because all of the mothers were recruited from the university community and all of them had relatively high levels of education (bachelor’s degree or above). Future research is needed to test the generalizability of the results among mothers with diverse educational backgrounds and those from different socioeconomic levels, as well as mothers from different cultures and regions.

## Conclusions

The present study advances our understanding of the neural mechanisms of reward processing in mothers. We observed mothers who exerted more effort to win rewards for their children than they exerted to win rewards for themselves and for charities, as reflected by their behavioral performance and by their brain’s responses during the anticipation and consumption phases. The results provide new evidence for the neural mechanisms of postpartum mothers’ bias toward their young children. Moreover, the source localization method provides compelling evidence for further understanding of the differences in neural activation between gaining rewards for mothers and for their children.
